# Stem Cell-Friendly Scaffold Biomaterials: Applications for Bone Tissue Engineering and Regenerative Medicine

**DOI:** 10.3389/fbioe.2020.598607

**Published:** 2020-12-14

**Authors:** Yongtao Zhang, Di Wu, Xia Zhao, Mikhail Pakvasa, Andrew Blake Tucker, Huaxiu Luo, Kevin H. Qin, Daniel A. Hu, Eric J. Wang, Alexander J. Li, Meng Zhang, Yukun Mao, Maya Sabharwal, Fang He, Changchun Niu, Hao Wang, Linjuan Huang, Deyao Shi, Qing Liu, Na Ni, Kai Fu, Connie Chen, William Wagstaff, Russell R. Reid, Aravind Athiviraham, Sherwin Ho, Michael J. Lee, Kelly Hynes, Jason Strelzow, Tong-Chuan He, Mostafa El Dafrawy

**Affiliations:** ^1^Department of Orthopaedic Surgery, The Affiliated Hospital of Qingdao University, Qingdao, China; ^2^Molecular Oncology Laboratory, Department of Orthopaedic Surgery and Rehabilitation Medicine, The University of Chicago Medical Center, Chicago, IL, United States; ^3^Ministry of Education Key Laboratory of Diagnostic Medicine, The School of Laboratory Medicine and the Affiliated Hospitals, Chongqing Medical University, Chongqing, China; ^4^Department of Burn and Plastic Surgery, West China Hospital of Sichuan University, Chengdu, China; ^5^Department of Orthopaedic Surgery, The First Affiliated Hospital of Guangzhou University of Chinese Medicine, Guangzhou, China; ^6^Departments of Orthopaedic Surgery and Neurosurgery, The Affiliated Zhongnan Hospital of Wuhan University, Wuhan, China; ^7^Department of Laboratory Diagnostic Medicine, The Affiliated Hospital of the University of Chinese Academy of Sciences, Chongqing General Hospital, Chongqing, China; ^8^Department of Orthopaedic Surgery, Union Hospital of Tongji Medical College, Huazhong University of Science and Technology, Wuhan, China; ^9^Department of Spine Surgery, Second Xiangya Hospital, Central South University, Changsha, China; ^10^Department of Surgery Section of Plastic and Reconstructive Surgery, The University of Chicago Medical Center, Chicago, IL, United States

**Keywords:** bone tissue engineering, biomaterials—cells, decellularizate ECM, polymer, stem cells—cartilage—bone marrow stem cells clinical application, mesenchymal stem cell

## Abstract

Bone is a dynamic organ with high regenerative potential and provides essential biological functions in the body, such as providing body mobility and protection of internal organs, regulating hematopoietic cell homeostasis, and serving as important mineral reservoir. Bone defects, which can be caused by trauma, cancer and bone disorders, pose formidable public health burdens. Even though autologous bone grafts, allografts, or xenografts have been used clinically, repairing large bone defects remains as a significant clinical challenge. Bone tissue engineering (BTE) emerged as a promising solution to overcome the limitations of autografts and allografts. Ideal bone tissue engineering is to induce bone regeneration through the synergistic integration of biomaterial scaffolds, bone progenitor cells, and bone-forming factors. Successful stem cell-based BTE requires a combination of abundant mesenchymal progenitors with osteogenic potential, suitable biofactors to drive osteogenic differentiation, and cell-friendly scaffold biomaterials. Thus, the crux of BTE lies within the use of cell-friendly biomaterials as scaffolds to overcome extensive bone defects. In this review, we focus on the biocompatibility and cell-friendly features of commonly used scaffold materials, including inorganic compound-based ceramics, natural polymers, synthetic polymers, decellularized extracellular matrix, and in many cases, composite scaffolds using the above existing biomaterials. It is conceivable that combinations of bioactive materials, progenitor cells, growth factors, functionalization techniques, and biomimetic scaffold designs, along with 3D bioprinting technology, will unleash a new era of complex BTE scaffolds tailored to patient-specific applications.

## Introduction

Considered as one of a few organs with high regenerative potential, bone is a dynamic form of connective tissue with complex cellular composition and highly specialized organic-inorganic architecture (Perez et al., 2018). Bone provides essential biological functions in the body, such as providing body mobility and protection of internal organs, regulating hematopoietic cell homeostasis, and serving as important mineral reservoir (Perez et al., 2018; Iaquinta et al., 2019). Repairing large bone defects represents a challenge for musculoskeletal surgeons. Each year, millions suffer from a decreased quality of life as a result of segmental bone defects caused by trauma, tumors, and other musculoskeletal pathologies (Dimitriou et al., 2011). Despite its remarkable remodeling potential of small defects, human bone tissue is unable to bridge and heal large segmental defects. Grafting materials are frequently required in segmental defects. In fact, autologous bone graft has been the gold standard for reconstruction of large bony deficiencies (Herford et al., 2011). However, use of autologous bone graft has several limitations, including donor site morbidity, its finite amount and limitation in terms of shape and structure. Allografts and xenografts may be the first alternatives to autografts, but they have the risk of transmission of infectious agents. Allografts may also be slow to incorporate or can even be rejected (Campana et al., 2014).

Bone tissue engineering (BTE) emerged as a promising solution to overcome the limitations of auto- and allografts. BTE combines concepts from bone biology and engineering techniques to design scaffolds enriched with stem cells to repair large bony defects (Bishop et al., 2017; Coalson et al., 2019; Qasim et al., 2019; Karakuzu-Ikizler et al., 2020; Wang et al., 2020a). However, bone tissue engineering practices have yet to be realized in clinical practice due to numerous limitations or lack of efficacy. Ideal bone tissue engineering is to induce bone regeneration through the synergistic integration of biomaterial scaffolds, bone progenitor cells, and bone-forming factors (Amini et al., 2012; Perez et al., 2018; Iaquinta et al., 2019). Thus, successful stem cell-based BTE would require a combination of abundant mesenchymal progenitors with osteogenic potential, suitable biofactors to drive osteogenic differentiation, and cell-friendly scaffold biomaterials (Bishop et al., 2017; Yan et al., 2018; Budsamongkol et al., 2019; Coalson et al., 2019; Mostafa et al., 2019; Qasim et al., 2019; Zhang et al., 2019; Pakvasa et al., 2021; Wang et al., 2020a).

Biomaterials represent the basic components of the scaffolds and serve a critical function in BTE. An ideal scaffold material should be biocompatible, biodegradable, osteoconductive, osteoinductive, and possess favorable mechanical properties (Babilotte et al., 2019; Lin Y. et al., 2019). Biomaterials used as scaffolds in BTE can be divided into the following categories: Ceramics, natural/synthetic polymers and decellularized extracellular matrix (dECM) or composites of the above (Kim et al., 2019; Rustom et al., 2019; Udomluck et al., 2019; Rothrauff and Tuan, 2020). The purpose of this review is to summarize the properties of these basic biomaterials used in BTE and their utilization in recent studies. We also discuss novel three-dimensional (3D) bioprinting technologies, the newest methods of scaffold fabrication, and 3D bioprinting applications in bone tissue engineering.

## Inorganic Compound-Based Ceramics

Ceramics are non-metallic inorganic compounds with a high level of stiffness and widely used as bone replacing biomaterials (Kaur et al., 2019). Ceramics can be divided into two categories: bioinert and bioactive. The bioinert ceramics, such as alumina and zirconia, induce immunoreaction and can form a thin fibrous layer at the bone interface (Ercal and Pekozer, 2020). Therefore, bioinert ceramics are not considered a usable material for bridging bone defects. On the other hand, bioactive ceramics have the ability of bone-bonding, also known as osteoconductivity. The main bioactive ceramics used as bone substitutes include hydroxyapatite (HA), β-tricalcium phosphate (β-TCP), biphasic calcium phosphate (BCP), whitlockite (WH), octacalcium phosphate (OCP), and bioglass (BG) (Table 1).

**Table 1:** Characteristics of various ceramics biomaterials used in BTE.

**Ceramics**	**Symbol**	**Formula**	**Advantage**	**Disadvantage**	**References**
Hydroxyapatite	HA	Ca_10_(PO4)_6_(OH)_2_	Osteoconduction Osteoinduction Biocompatibility	Lower biodegradability Poor mechanical properties	Shue et al., 2012; Tuukkanen and Nakamura, 2017; Jeong et al., 2019; Xiao et al., 2020
β-tricalcium phosphate	B-TCP	Ca_3_(PO4)_2_	Osteoconduction Osteoinduction Biocompatibility	Higher biodegradability Poor mechanical properties	Neo et al., 1993; Shue et al., 2012; Kim et al., 2014; Singh et al., 2016; Lu et al., 2019
Biphasic calcium phosphate	BCP	Mixture of HA and TCP	Osteoconduction Osteoinduction Biocompatibility Proper biodegradability	Poor mechanical properties	Tortelli and Cancedda, 2009; Shui et al., 2014; Bouler et al., 2017; Ebrahimi et al., 2017; Li et al., 2019
Whitlockite	WH	Ca_9_Mg(HPO_4_)(PO_4_)_6_	Osteoconduction Osteoinduction Biocompatibility Mechanical properties Contain magnesium ion	Synthesize difficultly	Jang et al., 2016; Kim et al., 2017b; Cheng et al., 2018; Jeong et al., 2019; Wang et al., 2020b
Octacalcium phosphate	OCP	(Ca_8_H_2_(PO_4_)_6_ 5H_2_O)	Osteoconduction Osteoinduction Biocompatibility	Higher biodegradability Innate brittleness makes it hard to sustain its shape	Kamakura et al., 1999; Dvorak et al., 2004; Bigi et al., 2005; Wang et al., 2015; Suzuki et al., 2020; Yanagisawa et al., 2020
Bioglass	BG	SiO_2_Na_2_OCaOP_2_O_5_	Osteoconduction Osteoinduction Biocompatibility	Lower biodegradability Poor mechanical properties	Wilson et al., 1981; Xynos et al., 2000; Baino et al., 2018; Kargozar et al., 2018

### Hydroxyapatite (HA): A Commonly Used Biosimilar Inorganic Matrix of Human Bone

HA is biochemically similar to the inorganic matrix of human bone, and is the most stable form of calcium phosphate due to its Ca/P molar ratio of 1.67 (Tuukkanen and Nakamura, 2017). HA is one of the most widely used biomaterials for both research and clinical applications (Shue et al., 2012). Its ideal biocompatibility is related to the ability to chemically bond bone. HA can be dissolved in body fluids and do not incite inflammatory reactions when applied clinically (Patel et al., 2002). HA biocompatibility is improved by promoting the osteogenic differentiation or the proliferation of mesenchymal stem cells (MSCs) and osteoblasts (Douglas et al., 2009). Studies have shown that HA is osteoconductive and osteoinductive (Xiao et al., 2020). Osteoconduction is the ability to promote bone growth on the surface of materials, whereas osteoinduction is the ability to induce progenitor cells into osteoblasts (Ambard and Mueninghoff, 2006). These bioactive characteristics are the result of the release of calcium and phosphate ions from the HA scaffold, which up-regulates the differentiation of osteoblasts, promotes bone formation through calcification, and increases osteoblastic differentiation gene markers (Jeong et al., 2019). Scaffolds composed of HA have no toxic effects on MSCs *in vitro*, and exhibit good biocompatibility and extensive osteoconductivity in rabbits *in vivo*, whereas the combination of MSCs with the scaffolds dramatically increased new bone formation (Wang et al., 2007).

Despite HA's many favorable characteristics as a bone biomaterial, it has some shortcomings including low degradability and poor mechanical properties (Siddiqui et al., 2018). As HA is one of the most stable forms of calcium phosphate, HA has been shown to degrade very slowly, even considered to be non-degradable (Nakamura et al., 2013). Its poor mechanical properties include low tensile strength and toughness, precluding its usage for high load-bearing applications (Siddiqui et al., 2018). As a result of its non-degradability and poor mechanical properties, HA is not used as a scaffold by itself. Instead, HA is used mainly in implant coating (Gustavsson et al., 2012). When used as an implant coating, HA improves osteoblast activity and increases the bony contact area (Darimont et al., 2002). Thus, in compound scaffolds HA is often mixed with polymers or tricalcium phosphate (TCP) to improve its degradability and mechanical properties (Dhivya et al., 2015).

### Tricalcium Phosphate (TCP): A Popular Osteoconductive/Osteoinductive Calcium/Phosphate Scaffold for BTE

TCP is another calcium phosphate biomaterial widely used in BTE. There are two available forms of TCP: α-TCP and β-TCP, both of which have a Ca/P molar ratio of 1.5 (Kim et al., 2017a,b). α-TCP has a monoclinic crystal structure while β-TCP has a rhombohedral crystal structure (Singh et al., 2016). Both can change into the other form at different temperatures (Horch et al., 2006). However, β-TCP is more widely used than α-TCP, because it is more stable and has a higher biodegradation rate (Kim et al., 2014). In addition, β-TCP has been shown to exhibit good biocompatibility in both *in vivo* and *in vitro* studies (Horch et al., 2006). β-TCP can bond to the host bone directly (Neo et al., 1993) and it increases the proliferation of osteoblasts and BMSCs (Yao et al., 2004). Furthermore, it promotes osteogenic differentiation of BMSCs (Neamat et al., 2009). Kamitakahara et al. also found that porous β-TCP scaffolds increase cell proliferation and adhesion when used in bone regeneration (Kamitakahara et al., 2008).

In recent years, β-TCP has been shown to have good osteoconductive and osteoinductive activity (Ogose et al., 2002). β-TCP can promote mineralization through calcium and phosphate ion release after degradation (Luginbuehl et al., 2010) and β-TCP scaffolds combined with bone morphogenetic proteins (BMP) can increase bone regeneration (Urist et al., 1987). Yuan et al. also reported new bone formation in the β-TCP scaffolds when implanted in dog muscles (Yuan et al., 2001).

Compared to HA, β-TCP is less stable but has a higher biodegradability, which allows newly formed bone to replace the β-TCP scaffold (Lu et al., 2019). β-TCP has two mechanisms of biodegradation: osteoclast-mediated and biological fluid-mediated biodegradation (Shue et al., 2012). However, the biodegradation rate of the β-TCP scaffolds is too fast to be compensated for by newly formed bone. Moreover, β-TCP scaffolds are brittle with a very low tensile strength (Ercal and Pekozer, 2020). Therefore, in order to counteract these drawbacks, other biomaterials are often mixed with β-TCP.

### Biphasic Calcium Phosphate (BCP): A Mixture of HA and β-TCP

The term BCP was first introduced in 1985 (Tortelli and Cancedda, 2009). BCP was considered a mixture of HA and β-TCP, although studies have shown that BCP is its own substance entirely rather than a combination with each of its component homogeneously mixed at the submicron level (Dorozhkin, 2012). BCP combines the advantages of HA and β-TCP, the biodegradability of β-TCP, and the biocompatibility, osteoconductivity and osteoinductivity of both (Bouler et al., 2017). The biodegradability of BCP depends on the HA/β-TCP ratio. A lower ratio results in a higher level of biodegradability (Bouler et al., 2017). Therefore, we can fabricate BCP with different levels of biodegradability by adjusting the HA/β-TCP ratio. As a result, there are over 30 commercially-used BCP bone substitution for various orthopedic applications (Ebrahimi et al., 2017).

Multiple studies demonstrated BCP's biocompatibility with osteoconductive and osteoinductive properties (Cordonnier et al., 2010). Cordonnier et al. found that MSCs adhered to BCP biomaterial and proliferated rapidly (Cordonnier et al., 2010). Li et al.'s study showed that BCP granules have good biocompatibility and can promote BMSCs adhesion, spread and proliferation (Li et al., 2019). Other studies reported that the osteoconductivity of BCP is most potent when its size ranges from 40 to 1,500 μm, and that BCP particles of this size could repair bone defects in animal models (Fellah et al., 2006). Li et al. found that porous BCP ceramic could up-regulate the osteogenic gene expression and promote osteogenesis when intramuscularly implanted in dogs (Li et al., 2019). Our previous study also showed that BCP scaffolds provide a cell-friendly environment to support the survival, propagation, and ultimately differentiation of BMP9-expressing osteoblastic progenitor cells (Shui et al., 2014).

### Whitlockite (WH): A Popular Calcium/Phosphate Scaffold Containing Magnesium Ions

Whitlockite (WH) is a calcium phosphate derivative containing magnesium ions in its lattice (Wang et al., 2020b). The Ca/P ratio of WH is 1.43 and it has a rhombohedral morphology similar to β-TCP (Jeong et al., 2019). Compared to HA and β-TCP, the biomechanical properties of WH are more favorable, especially its compressive strength (Jang et al., 2016; Cheng et al., 2018). WH is the second most abundant component in human bone occupying ~20% of bone mineral by weight (Kim et al., 2017b). WH is relatively rare in nature, indicating it is difficult to synthesize a pure phase of WH in a physiological system (Kim et al., 2017b; Cheng et al., 2018). Recently, studies found that WH could be easily synthesized in low-temperature conditions with useful characteristics as a scaffold in BTE (Jang et al., 2015; Kim et al., 2017b; Cheng et al., 2018).

It has been shown that the number of pits induced by osteoclasts on WH scaffold was two times higher than the number of pits induced by osteoclasts on an HA scaffold under the same conditions, rendering WH more biodegradable than HA (Kim et al., 2017b). In addition, WH has a higher solubility than HA in physiological conditions with a continuous release of magnesium and phosphate ions, enhancing the osteogenic activity of stem cells (Kim et al., 2017b; Cheng et al., 2018). When MSCs are cultured in HA/WH scaffolds with different ratios, cellular viability and proliferation were both higher than when they were cultured in HA alone (Cheng et al., 2018), although it was also reported that the cell viability of murine MSCs seeded across either WH or HA scaffolds was equal between the two scaffold environments (Jang et al., 2016). WH has been shown to poss superior osteoconductivity and osteoinductivity as human MSCs cultured on WH scaffolds exhibited significantly increased osteoblast-related gene expression compared to MSCs cultured on an HA scaffold, and WH-embedded cryogel had a higher mineral deposition compared to HA-embedded cryogel (Kim et al., 2017b). Nonetheless, HA and WH may have a synergistic effect on the promotion of growth and osteogenic activity of MSCs (Cheng et al., 2018). Thus, WH is expected to contribute to future research in BTE as a biomaterial due to its high osteogenic activity.

### Octacalcium Phosphate (OCP): A Calcium/Phosphate Scaffold in the Form of HA Precursor

Octacalcium Phosphate (OCP) is a calcium phosphate material and the precursor phase of HA formation. The Ca/P molar ratios of stoichiometric OCP is 1.33 (Yanagisawa et al., 2020). OCP has certain bone regenerative properties superior to HA including osteoinductivity, osteoconductivity, and biodegradability (Yanagisawa et al., 2020). Although OCP has many suitable properties as a bone substitute, its innate brittleness results in difficulty in sustaining its shape and therefore limits its clinical applications. Nonetheless, OCP combined with natural polymers is explored to treat bone defects (Suzuki et al., 2020).

Similar to other biocompatible calcium phosphate materials, the main components of OCP are calcium and phosphate ions (Wang et al., 2015). Bigi et al. found that when human primary osteoblasts were cultured on OCP coatings, the cells showed normal morphology with high rate of proliferation and viability (Bigi et al., 2005). Shelton et al. showed that when rat BMSCs were cultured on OCP scaffolds, cell number increased 40-fold after 20 days (Shelton et al., 2006). OCP is a bioresorbable ceramic and will degrade gradually in a physiological environment (Wang et al., 2015). OCP in a granule form degraded more rapidly than HA when implanted in the subperiosteal area of mouse calvaria (Kikawa et al., 2009). Moreover, recent studies demonstrated that the biodegradability of OCP occurred through osteoclastic cellular resorption, which in turn was enhanced by the intrinsic properties of OCP (Bigi et al., 2005; Suzuki, 2010).

OCP has better osteoconductivity and osteoinductivity than both HA and β-TCP (Kamakura et al., 1999). Calcium and phosphate ions are diffused from the surface of OCP during OCP–HA conversion (Suzuki, 2010). It has been shown that high concentrations of calcium and phosphate ions regulated osteoblastic cell differentiation (Dvorak et al., 2004), as it was reported that when mouse BMSCs cultured on a plate coated with OCP or HA, OCP was superior in inducing osteoblastic differentiation in a dose-dependent manner (Anada et al., 2008), and that OCP granules were able to repair critical-sized bone defects (Honda et al., 2009).

### Bioglass (BG): A Synthesized Ceramic Scaffold for BTE

BG is a form of bioactive ceramic widely used as scaffolds in BTE. The first form of BG (45S5) was created by Larry Hench in 1969 (Baino et al., 2018). Initially, Hench and his coworkers designed different glass formulations based on the SiO_2_-Na_2_O–CaO–P_2_O_5_ oxide system, and found that the composition 45SiO_2_-24.5Na_2_O−24.5CaO−6P_2_O_5_ (wt %) made the surface of the material reactive in physiological environments (Wilson et al., 1981), which was thus designated as 45S5. The 45S5 BG is the first example of the third generation biomaterial, which can induce genetic activation of specific cell pathways due to its released ionic dissolution products (Xynos et al., 2000). Over the past 40 years, many new types of BG have been proposed for use in specific BTE clinical applications (Rottensteiner-Brandl et al., 2018). In fact, from 1985 to 2016 the 45S5 BG-based scaffolds have been implanted in approximately 1.5 million patients to repair bone and dental defects (Baino et al., 2018).

The overall mechanism underlying BG degradation involves ion release and a surface reaction. First, sodium ions in the BG and hydrogen ions in the environment exchange and breakdown Si-O-Si bonds, leading to the formation of Si-OH bonds on the surface of the BG. The Si-OH bonds condense and form a SiO2 layer on the BG surface. The calcium and phosphate ions then move to the top of the newly formed SiO2 layer to form a new calcium-phosphate-rich layer (Piattelli et al., 2000). Lastly, hydroxyl carbonate apatite crystals form in the calcium-phosphate-rich layer, which is stoichiometrically equivalent to human bone (Johnson et al., 1997; Piattelli et al., 2000).

BG scaffolds exhibit good biocompatibility (Yang et al., 2014a) as osteoblast and multinucleated cells were shown to proliferate around BG particles implanted in a rabbit bone defect for 4 weeks, and mature bone was formed around the BG particles, and no gaps were seen at the bone-BG interface (Piattelli et al., 2000). It was reported that chitosan/BG 3D porous scaffolds exhibited good biocompatibility with high cell proliferation rates, and potential for BTE (Yang et al., 2014a). It has been shown that BG exhibited increased hemostasis, excellent handling properties, and tissue response with no adverse cellular reactions (Johnson et al., 1997). Furthermore, BG is osteoconductive, forming a stable bond to living bone, as well as osteoinductive, stimulating a path of regeneration and self-repair (Kargozar et al., 2018). Thus, BG has been used to augment bony defects at sites of dental implants (Johnson et al., 1997) and functions as both a filler and a coating material for implants in bone cavities (Turunen et al., 1997).

## Natural Polymers: Organic Biomaterial Extracted from Organisms

### Both Natural and Synthetic Polymers Are Commonly Used as Bone Biomaterials

Polymeric materials are easier to fabricate and mold into the desired shape and size (Hosseinpour et al., 2017). Natural polymers are intrinsically biocompatible and biodegradable and hence readily suited for tissue engineering applications, and in fact many kinds of polymeric materials are widely used in BTE (Müllner, 2019). Natural polymers commonly used in BTE include chitosan, collagen, gelatin, silk, alginate, and hyaluronic acid (Table 2) (Murphy et al., 2013; Ramesh et al., 2018). Synthetic polymers have increased longevity compared to natural polymers in terms of shelf life. Furthermore, they can be processed and fabricated in order to meet specific mechanical requirements. A number of synthetic polymers have been employed for BTE, including poly (lactic acid) (PLA), poly (glycolic acid) (PGA), poly (lactic-coglycolic acid) (PLGA), poly-ε-caprolactone (PCL), and poly(polyethylene glycol citrate-coN-isopropylacrylamide) (PPCN) (Table 3) (Murphy et al., 2013; Ramesh et al., 2018).

**Table 2:** Characteristics of various natural polymers used in BTE.

**Biomaterials**	**Characteristics**	**Advantages**	**Disadvantages**	**References**
Chitosan	Extracted from crustaceans Polysaccharide with positive charge Controlled biodegradability	Biocompatibility Osteoconduction Osteoinduction Resistance to bacteria	Low solubility Poor mechanical properties	Murphy et al., 2013; He et al., 2017; Preethi Soundarya et al., 2018; Zhao et al., 2019
Collagen	Important part of natural bone organic materials Most widely used biomaterials in BTE	Biocompatibility Biodegradability Osteoconduction Osteoinduction Easy processing	Low solubility Poor mechanism properties	Ferreira et al., 2012; Elango et al., 2016; Busra and Lokanathan, 2019; Müllner, 2019
Gelatin	Denaturalized collagen An ideal carrier of proteins, growth factors, and so on	Biocompatibility Biodegradability Osteoconduction High water-solubility Cell adhesion	Poor mechanism properties	Anjana et al., 2016; Kuttappan et al., 2016; Echave et al., 2019; Mahmoudi Saber, 2019
Silk	Produced from silkworms, bees, spiders, mites, and scorpions	Excellent mechanical properties Biocompatibility Controlled biodegradability	Low osteogenic capacity	Murab et al., 2013; Bhattacharjee et al., 2017; Nguyen et al., 2019
Alginate	Polysaccharide with negative charge Produced from brown seaweed	Biocompatibility High abundance resources Low prices Regulation of the inflammatory chemokines, and so on	Low biodegradability	Pravdyuk et al., 2013; Venkatesan et al., 2014; Sharmila et al., 2020
Hyaluronic acid	Glycosaminoglycan with negative charge Isolated from all the living organisms, mainly in the connective tissues and load bearing joints	Biocompatibility Osteoinduction Elasticity Water solubility	Low mechanical properties Rapid enzymatic degradation	Nguyen and Lee, 2014; Cui et al., 2015; Hemshekhar et al., 2016; Zhao et al., 2016; Ramesh et al., 2018

**Table 3:** Characteristics of various synthetic polymers used in BTE.

**Biomaterials**	**Acronym**	**Characteristics**	**Advantages**	**Disadvantages**	**References**
Poly (lactic acid)	PLA	Polyesterification reaction production of lactic acid Four different forms	Biocompatibility Biodegradability Sufficient mechanical properties Thermal stability	Poor cell adhesion Poor osteoinduction	Ramot et al., 2016; Tyler et al., 2016; Gregor et al., 2017; Ren et al., 2017; Gremare et al., 2018; Ramesh et al., 2018
Poly (glycolic acid)	PGA	Semicrystalline polymer Insoluble in most organic solvents	Biocompatibility Composited with other biomaterials	Poor cell adhesion Fast biodegradability Low mechanical properties Poor osteoinduction	Ulery et al., 2011; Asti and Gioglio, 2014; Ceccarelli et al., 2017; Cojocaru et al., 2020
Poly (lactic-coglycolicacid)	PLGA	A linear copolymer that combines PLA and PGA Delivery systems for drugs and therapeutic biomolecules	Biocompatibility Biodegradability Controllable mechanical properties Easily processed	Poor mechanical properties Poor osteoinduction Poor cell adhesion	Boukari et al., 2017; Zhao et al., 2017; Donnaloja et al., 2020; Essa et al., 2020
Poly-ε-caprolactone	PCL	Excellent crystallinity An crosslink *in situ* and print by injection	Biocompatibility Osteoinduction Osteoconduction Excellent mechanical properties	Low biodegradability Poor hydrophilicity	Lei et al., 2012; Kim and Kim, 2015; Sharifi et al., 2018; Lin W. C. et al., 2019; Dwivedi et al., 2020
Poly(polyethylene glycolcitrate-coN-isopropylacrylamide)	PPCN	Produced from poly(N-isopropylacrylamde) Thermosensitivity	Biocompatibility Biodegradability Osteoinduction Good solubility Antioxidant	Poor mechanical properties	Ohya et al., 2004; Yang et al., 2014b; Dumanian et al., 2017; Lanzalaco and Armelin, 2017; Zhao et al., 2018

### Chitosan: The Most Abundant Natural Amino Polysaccharide Biopolymer

Chitosan is mainly extracted from crustaceans (shrimps, crabs, lobsters, etc.), representing the most abundant natural amino polysaccharide and a versatile natural biopolymer formed by partial deacetylation of chitin under alkaline conditions (Younes and Rinaudo, 2015). Chitosan has the same structure as glycosaminoglycans, one of the components of the extracellular matrix critical for cell-cell adhesion. Thanks to its favorable properties including biocompatibility, biodegradability and anti-bacterial effects, chitosan-based scaffolds are used extensively in biomedical applications (LogithKumar et al., 2016; Balagangadharan et al., 2017; Preethi Soundarya et al., 2018). However, the mechanical strength of chitosan is low; therefore, other natural or synthetic polymers, metals and ceramics are added to chitosan to enhance its mechanical strength (Azzopardi et al., 2010). Nonetheless, chitosan can promote cell adhesion, proliferation, and differentiation (Jin et al., 2012; Dinescu et al., 2014). It was reported that, when adipose-derived MSCs were cultured on chitosan/silk nanofiber scaffolds, cell viability was enhanced (Chen et al., 2018), and that chitosan-based injectable and thermosensitive hydrogels improved the viability and proliferative activity of cultured mouse BMSCs at 7 days (Yan et al., 2010).

Chitosan is degraded into non-toxic absorbable small molecular amino acids and polysaccharides by chitosanolytic enzymes (Cheng et al., 2019). Chitosan modifications (e.g., covalent crosslinking and thiolation) can alter its degradation profile (Rottensteiner-Brandl et al., 2018). Chitosan scaffolds exhibit high cellular affinity for stem cells and promote stem cell osteogenesis as it was reported that chitosan-based nanofibrous scaffolds could mimic the extracellular matrix and recruit stem cells gathering to the scaffolds to facilitate seed cell adhesion, proliferation, and osteogenic differentiation *in vitro* (Cheng et al., 2014), and induced expression of osteogenic genes, ALP activity, and calcium deposition of stem cells (He et al., 2017). Chitosan not only promotes osteoblastogenesis but also reduces osteoclastogenesis as chitosan was shown to improve healing of fractures with greater trabecular bone formation in mice (Tan et al., 2014). We recently demonstrated that a pH-triggered, self-assembled, and bioprintable hybrid hydrogel scaffold composited with carboxymethyl chitosan and amorphous calcium phosphate exhibited excellent biocompatibility and promoted bone formation *in vivo* (Zhao et al., 2019). Chitosan has also been found to affect the activation of osteogenesis-related signaling pathways and to promote mineralization to facilitate bone regeneration via stimulation of trabecular bone production through the upregulation of the Runx2/osteocalcin/ALP signaling pathway in C57LB/6 mice (Ho et al., 2015).

### Collagen: The Most Common Component of Extracellular Bone Matrix

Collagen is the most abundant and widely distributed protein, constituting more than one-third of body protein tissue by weight (Preethi Soundarya et al., 2018). Approximately 29 types of collagen have been identified thus far. Among these, types 1, 2, 3, 5, and 9 collagen are known to form fibers and type 1 collagen is the most prevalent type in the ECM, especially in bone (Ferreira et al., 2012). Collagen protein has a complex hierarchical conformation, which is divided into four structures and is composed of 25 different alpha chains with each alpha chain composed of repeating units of Gly-X-Y. The X and Y residues are proline or hydroxyproline. Hydroxyproline is unique to collagen as it constitutes more than 50% of the total amino acid content. The fibrils are the final quaternary structure of the collagen protein (Ferreira et al., 2012). Collagen has been among the most widely used biomaterials in BTE thanks to its abundance and excellent properties, including biocompatibility, low antigenicity, absorbability, and easy processing (Nocera et al., 2018; Busra and Lokanathan, 2019).

Collagen can be processed into different physical forms, including injectable hydrogels, membranes and films, sponges and scaffolds, as well as micro-and nanospheres using a wide range of fabrication technologies to aid in targeted delivery, biocompatibility, stem cell differentiation and osteogenesis (Hu et al., 2008; Mencía Castaño et al., 2019). The chitosan–collagen materials promoted cell spread and proliferation (Wang and Stegemann, 2010). Collagen has also been utilized in guided bone regeneration procedures due to their biodegradability, osteoconduction, and osteoinduction (Ferreira et al., 2012). Collagen scaffolds provide an ideal environment for bone formation (Wang et al., 2016). They also serve as the best carrier for targeted delivery of small molecules such as antagonists of miR-16, which increases the relative levels of Runx2 and osteocalcin, thereby promoting mineral calcium deposition (Mencía Castaño et al., 2019). 3D nanofiber collagen scaffolds were shown to promote the proliferation of MC3T3-E1 cells, the expression of osteogenesis genes, ALP activity, and calcified matrix formation (Lee and Kim, 2018). However, the poor mechanical properties and low stiffness of collagen limit its solo applications in BTE (Ferreira et al., 2012) despite its favorable biocompatibility, biodegradation, and osteogenic properties (Elango et al., 2016).

### Gelatin: A Collagen Degradation Product for Cell Adhesion, Differentiation, and Proliferation

Gelatin is a product of collagen degradation and has the RGD (arginine, glycine, and aspartate) binding sequence. It is one of the most versatile naturally occurring biopolymers and widely used both individually and as a mixture in BTE owing to its ability to promote cell adhesion, differentiation, and proliferation (Mahmoudi Saber, 2019). As a natural origin polymer, gelatin holds several advantages over its precursor, collagen, such as low water solubility, one of the main limitations of collagen as a biomaterial (Kolesky et al., 2016; Echave et al., 2019). Gelatin can be enzymatically degraded into its respective amino acids and cause no harm to the body or cells (Mahmoudi Saber, 2019). In addition, gelatin can create poly-ionic complexes with different therapeutic factors such as proteins, growth factors, nucleotides, and polysaccharides (Echave et al., 2019).

The composite scaffold of HA/gelatin is potentially useful for hard-tissue regeneration (Kuttappan et al., 2016). A gelatin-siloxane scaffold showed ideal cytocompatibility, while also stimulating osteoblast proliferation and differentiation *in vitro* (Ren et al., 2002), while when siloxane-calcium silicate was modified by gelatin, it showed good biocompatibility and osteoconductivity, as well as robust bone formation capability as tested by implanting the scaffold into rat calvarial defects (Kuttappan et al., 2016). It was also reported that multi-sized porous β-TCP scaffolds coated with 3% gelatin under a vacuum displayed enhanced compressive strength as well as increased cell differentiation in comparison to their non-coated counterparts (Kim et al., 2012). Furthermore, gelatin-calcium phosphate nanofibers, composed of β-TCP nanoparticles incorporated into electropunk gelatin nanofibers, improved proliferation and osteogenic differentiation of rat MSCs compared to pure calcium phosphate nanofibers (Kuttappan et al., 2016). Lastly, gelatin is also an ideal carrier for proteins and various growth factors (Krishnan et al., 2015; Anjana et al., 2016). For example, Gelatin/HA fibrous scaffold with platelet-rich plasma gel was able to induce the differentiation of MSCs into an osteogenic cell line (Anjana et al., 2016).

### Silk: A Biopolymer With Excellent Mechanical Properties Derived From Silkworms, Bees, Spiders, Mites, and Scorpions

Silk is a naturally occurring protein biopolymer produced by silkworms, bees, spiders, mites, and scorpions although the silk from each species is tailored to their specific evolutionary needs (Bhattacharjee et al., 2017). Regardless of source, silks are primarily composed of two types of proteins namely silk fibroin (fibrous) and sericin (globular). Silk fibroin makes up the fibrous part of the filament and is structurally composed of heavy chains and light chains, whereas sericin is a water-soluble, glue-like protein that coats the two chains of fibroin and constitutes about 25–30% of the weight of silk fibroin (Nguyen et al., 2019). Silk protein is a promising biomaterial in BTE owing to its excellent mechanical properties and biocompatibility (Midha et al., 2016). Silk can also be molded easily into various forms of scaffolds (Murab et al., 2013), hydrogels (Murab et al., 2015), and composites (Sun et al., 2012).

The biodegradability of silk biomaterial can be controlled by modulating its structures including chain length and secondary conformations (Bhattacharjee et al., 2017). Silk fiber processing solvents can affect the secondary structure and biodegradability of silk. Organic solvent-based silk takes a year or more to get completely absorbed *in vivo* (Zhang et al., 2009), while aqueous solvent-based silk only requires a maximum of 6 months to get completely absorbed *in vivo* (Li S. et al., 2018). This property is helpful in repairing critical bone defects as silk scaffolds can provide sufficient structural support until native bone is completely regenerated (Mandal and Kundu, 2009).

The products of silk degradation are glycine and alanine, which can be reused to synthesize new proteins *in vivo* (Bhattacharjee et al., 2017). Hence, silk biomaterials have excellent biocompatibility and a low risk of triggering immunogenic reactions (Bhattacharjee et al., 2017; Du et al., 2019; Sartika et al., 2020). A pure 3D silk scaffold that was made using a lyophilization method demonstrated superior biocompatibility *in vitro* (Sartika et al., 2020). Although the low osteogenic potential of silk limits its applications in BTE, in combination with other biomaterials, silk can enhance bone regeneration (Lee et al., 2017). When MSCs were cultured on a silk/HA scaffold, such scaffold showed an increase in ALP activity and mineralization after 12 days (Farokhi et al., 2018).

### Alginate: A Marine Anionic Biopolymer From Brown Seaweeds for BTE

Alginate is the most abundant marine anionic biopolymer, which is produced industrially from brown seaweeds such as *Laminaria hyperborea, Laminaria digitata, Laminaria japonica, Ascophyllum nodosum*, and *Macrocystis pyrifera*. The primary source of alginate is from the cell walls and intracellular spaces of brown seaweed (Venkatesan et al., 2014). It is composed of guluronic acid (G) and mannuronic acid (M). The composition (G/M ratio), sequence, and molecular weight are critical factors affecting the physical properties of alginate (George and Abraham, 2006). The alginate molecules are necessary for plant growth in the sea because they provide the plant with both flexibility, and strength. The attractive properties of Alginate include biocompatibility, non-toxicity, non-immunity, and biodegradability in addition to its high abundance with a low cost. Chemical and physical modifications of alginate can fine tune its properties and functions, such as biodegradability, mechanical strength, gelation property, and cell affinity, toward respective applications. Furthermore, alginate can be easily formed into a variety of hydrogels, microspheres, microcapsules, sponges, foams, and fibers (Sharmila et al., 2020). Therefore, alginate and its composites can be prepared through various crosslinking methods and are widely used as a biomaterial in BTE, next to chitosan among the natural polysaccharides (Sharmila et al., 2020).

The biocompatibility of alginate scaffolds has been measured with various types of cells *in vitro* (Pravdyuk et al., 2013). When human MSCs were encapsulated in alginate microspheres and then cryopreserved, the viability of MSCs and their metabolic rates were maintained (Pravdyuk et al., 2013). Alginate-based scaffolds also showed favorable biocompatibility (Florczyk et al., 2011; Sharmila et al., 2020). For example, a porous chitosan-alginate scaffold was shown to enhanced cell proliferation (Florczyk et al., 2011).

Alginate scaffolds possess bone regeneration properties (Hwang et al., 2009; Kim et al., 2020), as embryonic stem cells encapsulated in alginate hydrogels cultured in a microgravity bioreactor, showed enhanced differentiation and osteogenesis (Hwang et al., 2009). HA mixed with alginate increased its mechanical strength, and the resulting scaffold possessed excellent osteogenic effects (Venkatesan et al., 2015). Injection of a thermosensitive alginate/β-TCP/aspirin scaffold with an interconnected porous microstructure into the periosteum of rat cranial bone promoted new bone formation (Fang et al., 2018). Furthermore, alginate was also used in combination with other biomaterials, such as collagen, gelatin, and bioglass, to increase its mechanical strength, cell adhesion, and osteogenic differentiation ability (Venkatesan et al., 2015).

### Hyaluronic Acid: A Hydrophilic Glycosaminoglycan for BTE

Hyaluronic acid is a hydrophilic linear unbranched glycosaminoglycan made up of alternating residues of d-glucuronic acid and N-acetyl glucosamine with highly variable length and molecular weight (up to 10^6^ Da) (Zhao et al., 2016). It can be isolated from the vitreous humor of cows, although it is found in almost all living organisms, mainly in the connective tissues and load bearing joints, etc. (Ramesh et al., 2018). Hyaluronic acid is the primary component of the extracellular matrix of connective tissues, and presented in high concentrations in the synovial joint fluids, vitreous of the eyes, hyaline cartilage, intervertebral disc nucleus, and umbilical cord (Hemshekhar et al., 2016). Hyaluronic acid plays important physiological roles, such as lubrication of arthritic joints, control of the viscoelastic properties of soft tissues and the motility, adhesion and organization of cells (Hemshekhar et al., 2016; Gokila et al., 2018). Hyaluronic acid is also involved in key signaling pathways and regulates cell proliferation, survival, motility, and differentiation (Toole, 2004). Furthermore, it has been shown that hyaluronic acid regulates inflammatory chemokines, metalloproteinases, and tissue inhibitors, which help form better scaffolds. Owing to its elasticity, biocompatibility, non-immunogenicity, water solubility, and osteoinductivity, hyaluronic acid is an excellent polymer of choice for BTE (Collins and Birkinshaw, 2013).

Hyaluronic acid scaffolds were shown to support the attachment and proliferation of MC3T3-E1 cells and could be degraded at acidic pH values or by hyaluronidase and reducing substances (Cui et al., 2015). The rapid enzymatic degradation of hyaluronic acid must be taken into account *in vivo*, potentially decreasing the mechanical properties of hyaluronic acid -based scaffolds (Drury and Mooney, 2003). Hyaluronic acid and gelatin (Gel) hydrogel loaded into a BCP ceramic created a new scaffold with highly interconnected porosity, with an average compressive strength of 2.8 ± 0.15 MPa, ideal as a cancellous bone substitute. This scaffold increased cell proliferation and ALP activity, and promoted new bone formation and mineralization, with delayed degradation within 3 months after implantation (Nguyen and Lee, 2014). Thus, hyaluronic acid has great potential as a scaffolding material in BTE.

## Synthetic Polymers: Artificially Synthesized Organic Biomaterials for BTE

### Poly (Lactic Acid) (PLA): A Thermally Stable Polyesterification Reaction Product of Lactic Acid Biomaterial for BTE

Poly (lactic acid) (PLA) is a product of polyesterification reaction of lactic acid, characterized by several features fundamental for bone regeneration, such as non-toxicity, biocompatibility, thermal stability, and biodegradability (Gregor et al., 2017). PLA has been approved by the FDA as a hydrophobic aliphatic polyester in different biomedical and clinical applications as it degrades to the physiological product lactic acid (Tyler et al., 2016). The thermal stability and biodegradability of PLA are dependent on the choice and distribution of stereoisomers within the polymer chains as well as molecular weights (Gremare et al., 2018). Therefore, there are four different forms of PLA, including poly (L-lactic acid) (PLLA), poly (D-lactic acid) (PDLA), Poly (D,L-lactic acid) (PDLLA), and meso-poly(lactic acid), which result in PLA with a broad range of physiochemical properties (Ramot et al., 2016).

PLA was successfully printed in scaffolds with different pore sizes, sufficient mechanical integrity, and biodegradability, and BMSCs cultured on the scaffold, were not affected in terms of metabolic activity and cell viability (Gremare et al., 2018). Osteosarcoma cells were also tested and indicated the PLA scaffold was non-cytotoxic and promoted cell growth, cell viability, and osteogenic gene expression (Velioglu et al., 2019). PLA-based composite materials with other polymers or ceramics were shown osteoconductive and osteoinductive (Ramesh et al., 2018). PLA/HA biocomposites showed good biocompatibility and osteo-differentiation toward osteoblast precursors with the increase in cell numbers, cell adhesion, and promotion of ALP activity (Kim et al., 2006). When BMSCs were seeded on a bent nanofibrous mesh scaffold containing PLA and gelatin (in a 1:1 in weight ratio), the mesh promoted osteogenic differentiation of BMSC sheets compared to the control, and the blended biomaterial increased bone formation within rat cranial defects (Ren et al., 2017).

### Poly (Glycolic Acid) (PGA): A Bioabsorbed Scaffold Material With a High Tensile Modulus

PGA is a semicrystalline polymer with a high tensile modulus and insoluble in most organic solvents. Owing to its high porosity, PGA allows diffusion of nutrients upon implantation and subsequent neovascularization (Ramesh et al., 2018). Moreover, PGA is easily handled and can be fabricated into different shapes (Asti and Gioglio, 2014). PGA was the first synthetic biodegradable polymer that was approved by the FDA for use in the production of absorbable sutures (Ramesh et al., 2018). However, PGA is absorbed rapidly (within 6 to 8 weeks), the degradation products, such as glycolic acid and other acidic products can evoke a strong inflammatory response, which limits its biomedical applications (Ulery et al., 2011). For this reason, PGA is not used alone but in the combination form of PLGA (Ceccarelli et al., 2017). Another disadvantage of PGA is its low mechanical strength. To overcome these drawbacks, PGA is usually used in a composite form with other biomaterials, such as HA (Dunne et al., 2010; Cojocaru et al., 2020). For example, Dunne et al. found that PGA/HA composite scaffolds had good biodegradability and the proper porosity required for cellular infiltration and attachment (Dunne et al., 2010). PGA-hyaluronan with high porosity was used in the three-dimensional culturing of meniscus cells, where the scaffold displayed good biocompatibility (Cojocaru et al., 2020).

### Poly (Lactic-Coglycolic Acid) (PLGA): A Linear Copolymer of PLA and PGA and Commonly-Used Synthetic Polymer Biomaterial for BTE

PLGA is a linear copolymer that combines PLA and PGA, and is a widely used synthetic polymer material in BTE. It has favorable properties as a biomaterial in BTE, including biodegradability, biocompatibility, controllable mechanical properties, and easy processing (Boukari et al., 2017). PLGA has also been approved by the FDA for use in clinical applications as it mitigates the disadvantages of both PLA and PGA (Lü et al., 2009). One appealing property of PLGA is that its degradation rate is tunable, ranging from weeks to months, via a variation of the ratio of the two monomers (Donnaloja et al., 2020). For example, PLGA containing 75% PGA is amorphous and hydrolytically unstable, thus degrading faster. PLGA is easily broken down *in vivo* by hydrolysis into lactic acid and glycolic acid, both of which are non-toxic monomers physiologically metabolized by the tricarboxylic acid cycle for final excretion in the lungs (Essa et al., 2020). In fact, PLGA is one of the most widely used synthetic polymers for drug delivery systems and therapeutic biomolecules due to its favorable biological properties (Ortega-Oller et al., 2015). Nonetheless, its suboptimal mechanical properties, poor osteoinductivity, and poor cell adhesion limit its usage in BTE.

In order to overcome the above shortcomings in BTE, PLGA often combines with other biomaterials, such as ceramics like bioglass, HA, TCP and natural polymers (Boukari et al., 2017; Zhao et al., 2017; Bharadwaz and Jayasuriya, 2020; Probst et al., 2020). For example, PLGA/HA composite fibrous scaffolds were fabricated by the melt-spinning method, in which BMP-2 was incorporated with the help of polydopamine (Zhao et al., 2017). The enhanced roughness of PLGA/HA fibrous scaffolds due to the polydopamine coat caused increased hydrophilic property, cell adhesion and proliferation of cells. Moreover, the composite scaffold increased bone mineral deposition and ALP activity (Zhao et al., 2017). PLGA/chitosan scaffolds were shown to have better mechanical properties and a much steadier drug release (Boukari et al., 2017). When human MSCs were cultured on this composite scaffold, cell proliferation increased over time and the extent of mineralization increased after 21 days (Boukari et al., 2017).

### Poly-ε-Caprolactone (PCL): A Synthetic Polymer With Alterable Mechanical Properties and a High Degree of Crystallinity

PCL is one of the most commonly used synthetic polymers in BTE. PCL has easily manipulable mechanical properties, a high degree of crystallinity, and suitable properties such as non-toxicity, biocompatibility, and biodegradability, leading to its use as an implantable polymer material approved by the FDA (Atyabi et al., 2016; Sharifi et al., 2018). Though biodegradable, PCL, especially in its high molecular weight form, has a slow degradation rate, restricting its usage in BTE. In fact, it degrades at the slowest rate among all polyesters and may require 3 years to completely eliminate itself from the host body, either by microorganisms or by hydrolysis of its aliphatic ester linkage (Shive and Anderson, 1997), although a PCL-based composite scaffold showed a faster degradation rate, such as bioactive glass microspheres reinforced to PCL (Lei et al., 2012).

The poor hydrophilic nature of PCL affects the cell attachment, proliferation and differentiation, posting the most important challenge to the initial step of cell culturing (Sharifi et al., 2018). To increase the hydrophilicity of PCL, its surface can be modified using several methods, such as coating the scaffold with composites of silk, gelatin, growth factors, and proteoglycans (Dwivedi et al., 2020). For example, a porous nanofibrous scaffold composed of HA, silk, and PCL was blended via a one-step emulsion electrospinning, and the resulting product exhibited hydrophilicity while promoting human primary skin fibroblast proliferation and filopodia protrusion (Li et al., 2012). The PCL/Cobalt/HA composite membrane increased osteoblast cell proliferation and calcium deposition production, compared with PCL only (Lin W. C. et al., 2019).

PCL scaffolds were shown to promote bone regeneration (Faia-Torres et al., 2015; Kim and Kim, 2015; Dwivedi et al., 2020). It was shown that PCL scaffolds with a surface roughness of 0.9–2.1 μm promoted the osteogenic ability of the hMSCs seeded on it when cultured in a dexamethasone-deprived osteogenic induction medium (Faia-Torres et al., 2015). Highly roughened PCL surfaces on an apatite layer using oxygen plasma-etching led to improved cell viability and osteogenic ability of MC3T3-E1 cells when soaked in simulated body fluid (Kim and Kim, 2015).

### Poly(Polyethylene Glycol Citrate-coN-Isopropylacrylamide): A Group of Thermoresponsive Synthetic Polymer Biomaterials for BTE

Poly(N-isopropylacrylamide) (PNIPAAm) is a thermoresponsive hydrogel used to copolymerize and graft synthetic polymers in the biomedical field (Lanzalaco and Armelin, 2017). PNIPAAm is well-known for its water solubility and its lower critical solution temperature that falls close to body temperature (around 36.5–37.5°C) (Lanzalaco and Armelin, 2017). Furthermore, PNIPAAm is highly biocompatible with animal cells (Ohya et al., 2004; Lanzalaco and Armelin, 2017). It was reported that the PNIPAAm/gelatin scaffold in the subcutaneous tissue of rat promoted fibroblast proliferation (Ohya et al., 2004). However, the poor biodegradability of PNIPAAm hydrogel has limited its applications. Several strategies for the preparation of biodegradable PNIPAAm-based hydrogels are being explored via mixing with poly-amino acids, polysaccharides, and poly-esters (Lanzalaco and Armelin, 2017). Another limitation for PNIPAAm-based materials is it may induce oxidative stress in tissue, which can contribute to chronic inflammation, leading to impaired wound healing and lowered the efficacy of cell-based therapies and medical devices.

To address the above challenges, a thermoresponsive macromolecule poly(polyethylene glycol citrateco-N-isopropylacrylamide) (PPCN), based on PNIPAAm, was developed. PPCN is a novel antioxidant which is both degradable and biocompatible (Yang et al., 2014b). The process of designing PPCN involves first synthesizing a water-soluble acrylated polydiolcitrate using citric acid, polyethylene glycol, and glycerol 1,3 diglycerolate diacrylate. Then, the water-soluble polydiolcitrate can be further functionalized with PNIPAAm to form a PPCN polymer (Yang et al., 2014b). PPCN has been proven to be an excellent biomaterial for bone formation as demonstrated in our laboratory (Dumanian et al., 2017; Morochnik et al., 2018; Zhao et al., 2018; Lee et al., 2019). We showed that when graphene oxide was incorporated into PPCN to fabricate the resultant hybrid materials referred to as GO-P, the composite scaffold maintained the thermoresponsive behavior of PPCN, effectively supporting BMSC survival and proliferation (Zhao et al., 2018). Furthermore, the GO-P scaffolds promoted BMP9-transduced BMSC mineralization and vascularized trabecular bone (Zhao et al., 2018). Mesenchymal progenitor immortalized murine adipocyte cells (iMADs) transduced with adenovirus expressing BMP-9 were cultured on a PPCN/gelatin scaffold to cure critical-sized cranial defects in mice. The scaffold promoted osteogenesis and exhibited significantly greater osteogenesis *in vivo* (Lee et al., 2019). Another study of murine-derived calvarial mesenchymal progenitor cells (iCALs) transduced by BMP-9 cultured on a PPCN/gelatin scaffold, to repair critical sized cranial defects, also promoted osseointegration and mature bone formation (Dumanian et al., 2017).

## Decellularized Extracellular Matrix (dECM) Provides a Complex Microenvironment of Native Tissue Biomaterials for BTE

Even though biomaterials may possess favorable properties such as biocompatibility, biodegradability, osteoconductivity, and osteoinductivity, those materials are unable to replicate the complex microenvironment of native bone tissue (Benders et al., 2013). While allografts and xenografts are ideal methods for reconstructing bone defects, they carry the risk of transmission of infectious agents. To improve the safe use of allo- and xenografts, decellularization techniques are used to produce acellular scaffolds or decellularized extracellular matrix (dECM) (Gentili and Cancedda, 2009; Pati et al., 2015). The dECM functions as the non-cellular component of tissue that provides the structural and functional molecules to determine the cell's fate and serve as potential therapeutic materials (Frantz et al., 2010).

The major components of ECM are collagen and proteoglycans that are secreted by resident cells and assembled in a manner specific to individual tissue types, leading to a specialized ECM for each tissue type. The ECM is a dynamic entity that consistently responds to external influences including biomechanics and hormones (Nelson and Bissell, 2006). Given the high complexity, inherent compositional specificity, and the ability to support cell growth and differentiation, the engineered scaffolds based on tissue-specific natural ECM are likely more suitable than those built from artificial compounds for bone tissue regeneration (Benders et al., 2013).

Decellularization is the first step in processing ECM for use in regenerative therapy preserving the complex biomolecular and physical cues of the ECM (Crapo et al., 2011). The foremost challenge of the decellularization process is maximal removal of cellular components while limiting damage to the ECM (Crapo et al., 2011). The specific methods of decellularization depend on the tissue types and usually involve a combination of physical, enzymatic, and chemical processes. Physical decellularization is the least disruptive method, and includes freezing-thawing cycles, osmotic pressure, hydrostatic pressure, sonication, and electroporation (Oliveira et al., 2013; Santoso et al., 2014). Chemical decellularization can be divided into two subcategories. The first is to treat tissues with acids or bases to promote cell degradation and remove cellular components such as nucleic acids (Petersen et al., 2012). The second is to use non-ionic, ionic, or zwitterionic detergents such as Triton X-100, sodium dodecyl sulfate (SDS), and 3-((3-cholamidopropyl) dimethylammonio)-1-propanesulfonate, respectively, to denature proteins (Calle et al., 2016). Enzymatic decellularization is the use of nucleases, like deoxyribonuclease, ribonuclease, proteases, and trypsin, to facilitate cell degradation and the removal of residual nuclear materials from the tissue after chemical decellularization (Crapo et al., 2011). Since each decellularization method alone cannot completely remove cellular debris from the tissue, a combination of three methods is usually employed (Lu et al., 2012).

The scaffold forms of dECM used in BTE demonstrated fine biocompatibility and osteogenic capacity maintaining original geometry, cell-laid matrix, hydrogel, and particles (Benders et al., 2013). Smith et al. used a biocompatible biological scaffold from ECM, prepared via a novel washing technique to remove marrow components, which increased biocompatibility, conserved biomechanical stability, and induced osteogenic differentiation of MSCs (Smith et al., 2015). The cell-laid matrix refers to a layer of dECM coating on scaffolds, initially secreted by cells seeded onto the scaffolds, then decellularized, and recellularized for use as a regenerative therapy (Kumar et al., 2016). Kumar et al. seeded osteoblasts to deposit mineralized ECM on PCL/HA scaffolds, which displayed higher cell adhesion, growth, motility, and osteogenic differentiation compared to bare PCL/HA scaffolds (Kumar et al., 2016). Thibault et al. seeded rat MSCs on electrospun PCL and then cultured the sample in a complete osteogenic medium to generate mineralized ECM. The cell-laid matrix scaffold triggered higher osteogenic differentiation of MSCs and calcium deposition compared to bare PCL scaffolds (Thibault et al., 2010).

Decellularized small intestinal submucosa (dSIS) for ECM ornamentation has also been investigated (Li M. et al., 2018). Li et al. seeded osteoblasts on a dSIS scaffold to generate an osteogenic and mineralized ECM construct (Os/M-ECM-dSIS), which had similar morphology and inorganic components compared to natural bone and a higher mechanical strength than ECM-SIS (Li M. et al., 2018). Furthermore, these scaffolds also enhanced cell adhesion, proliferation, and osteogenic differentiation. In a calvarial defect model, new bone formation was enhanced in defects implanted with the Os/M-ECM-SIS scaffolds compared with ECM-SIS scaffolds via the Bmp/Smad-signaling pathway (Li M. et al., 2018). The dECM hydrogel was also created from solubilized dECM digested with pepsin. Since the dECM is digested into a homogenized and solubilized biomaterial, it does not preserve the architecture of the natural ECM (Sawkins et al., 2013). In terms of bone dECM, the production of a homogenized and solubilized biomaterial requires demineralization without digestion. A thermally responsive hydrogel made from both demineralized and decellularized bovine bone ECM was shown to promote the growth of primary calvarial cells in mice (Sawkins et al., 2013). Another novel bone dECM hydrogel scaffold combined with alginate incorporating human BMSCs and BMP2 was injected between segmental defects of embryonic chicken femurs and cultured *ex vivo* for 10 days, showing that the scaffold augmented skeletal tissue formation (Smith et al., 2014).

The bone dECM can be milled into particles that contain bone ECM components, which can be further combined with other biomaterials for use in BTE (Townsend et al., 2017; Beachley et al., 2018). Townsend et al. combined HA, decellularized cartilage, and hyaluronic acid to form colloidal gels that promoted bone regeneration when used in the treatment of bone defects *in vivo* (Townsend et al., 2017). Beachley et al. found a dECM particle-based composite hydrogel could be formulated by using modified GAGs (chondroitin sulfate, hyaluronic acid) that covalently bind to tissue particles; and bone dECM particles. The dECM particle-based hydrogel demonstrated enhanced bone formation compared to hydrogels that did not contain dECM particles (Beachley et al., 2018). Hung et al. created novel 3D printed hybrid dECM/PCL scaffolds by combining the bone dECM particles with PCL for bone regeneration. The scaffolds displayed robust mechanical properties, enhanced cell adhesion, upregulated osteogenic differentiation, generated greater bone volume *in vivo*, and increased calcification, compared with PCL alone (Hung et al., 2016).

Overall, dECM is a tissue-derived biomaterial that can be used as a bioactive component for tissue engineering applications. The addition of bone dECM frequently exhibited enhanced bone regenerative capabilities and guided the osteogenic differentiation of seeded stem cells even without the addition of exogenous growth factors. However, many improvements have to be made for the use of dECM in standard clinical treatments, such as methods of dECM tissue processing, tissue sources, and storage conditions. Therefore, the utilization of dECM in tissue engineering applications is still in development and requires more research, especially in exploring the best decellularization methods.

## Conclusions and Future Directions

BTE is regarded as the most promising solution for critical-sized bone defects. The basic subunit in BTE is the scaffold, which provides a site for cell attachment, proliferation, and differentiation, as well as providing mechanical strength. Biomaterial selection and scaffold fabrication techniques are the two most important aspects to achieve these goals. Although the biomaterials discussed in this review have many advantages as scaffold materials in BTE, there are also shortcomings that limit their applications when used on their own. Therefore, discovery of new materials and manufacturing of composite scaffolds using these existing biomaterials will still constitute the center of future research efforts in the field of BTE. When combined with the developing array of bioactive materials, growth factors, functionalization techniques, and biomimetic scaffold designs, together with 3D bioprinting technology, the potential to create complex BTE scaffolds tailored to patient-specific applications in the future is vast.

## Author Contributions

All authors contributed significantly to the work and have read and concurred with the content of the manuscript.

## Conflict of Interest

The authors declare that the research was conducted in the absence of any commercial or financial relationships that could be construed as a potential conflict of interest.
